# Neck pain and anxiety do not always go together

**DOI:** 10.1186/1746-1340-18-6

**Published:** 2010-03-11

**Authors:** Corrie Myburgh, Kirsten K Roessler, Anders H Larsen, Jan Hartvigsen

**Affiliations:** 1 University of Southern Denmark, Institute of Clinical Biomechanics, Campusvej 55, Odense M, 5230, Denmark; 2 Odense University Hospital, Sdr Boulevard 29, 5000 Odense C, Denmark; 3 Nordic Institute of Chiropractic and Clinical Biomechanics, Forskerparken 10, DK-5230, Odense M, Denmark

## Abstract

Chronic pain and psychosocial distress are generally thought to be associated in chronic musculoskeletal disorders such as non-specific neck pain. However, it is unclear whether a raised level of anxiety is necessarily a feature of longstanding, intense pain amongst patient and general population sub-groups. In a cohort of 70 self-selected female, non-specific neck pain sufferers, we observed relatively high levels of self-reported pain of 4.46 (measured on the 11 point numerical pain rating scale (NRS-101)) and a longstanding duration of symptoms (156 days/year). However, the mean anxiety scores observed (5.49), fell well below the clinically relevant threshold of 21 required by the Beck Anxiety Inventory. The cohort was stratified to further distinguish individuals with higher pain intensity (NRS>6) and longer symptom duration (>90 days). Although a highly statistically significant difference (p = 0.000) was subsequently observed with respect to pain intensity, in the resulting sub-groups, none such a difference was noted with respect to anxiety levels. Our results indicate that chronic, intense pain and anxiety do not always appear to be related. Explanations for these findings may include that anxiety is not triggered in socially functional individuals, that individual coping strategies have come into play or in some instances that a psychological disorder like alexithymia could be a confounder. More studies are needed to clarify the specific role of anxiety in chronic non-specific musculoskeletal pain before general evidence-driven clinical extrapolations can be made.

## Findings

The influence of anxiety as a psychosocial factor relevant to body pain has been widely investigated [1-5]. With respect to chronic neck[1,6] and low back[4] research, in particular, a varying role for anxiety has been observed. Depending on factors such as whether anxiety is considered independently or as a part of psychosocial distress and the type of methodology followed, its importance varies between a co-morbidity and episode trigger. However, in general, it appears that anxiety is thought to be associated with intensity and duration of pain[6]. Specifically, chronic pain sufferers with high levels of pain are considered likely to reveal raised levels of anxiety with respect to their condition/symptoms[4,7].

Recently, it has been postulated that this relationship may extend to cases where chronic, regional pain is identified in combination with Fibromyalgia or Myofascial Pain Syndrome (MPS)[3,4]. However, as it is currently still unclear whether anxiety is necessarily always associated with chronic pain or whether there are instances when persons with longstanding and/or intense pain in fact exhibit low levels of anxiety, this may be a premature clinical extrapolation.

## Aim

To report anxiety levels in relation to neck pain intensity and duration in a self-selected group of Danish female subjects presenting with non-specific neck pain.

## Methods

The sampling protocol was part of a myofascial trigger point (TP) inter-examiner reliability study where a random case mix of symptomatic and asymptomatic subjects was as required. Female subjects between the ages of 20-45 years old who performed at least 4 hours of office work per day and reported neck pain in the region of the upper Trapezius muscle were sought[8]. After an initial telephonee screening, subjects completed an electronic questionnaire. Outcomes solicited included subjective pain rating (NRS-101)[9], the Beck Anxiety Inventory (BAI)[10] and the Standardized Nordic Pain Questionnaire[11]. Regardless of symptom state, individuals were evaluated by an index clinician, who acquired further anthropometric data and rated the likelyhood of subjects harbouring diagnostically relevant trigger points (TPs) based on their historical information. Subjects were then sent on for physical examination of the neck/shoulder region. Ethical clearance was granted through the local ethics committee (Region of Southern Denmark). In order to stratify and compared the data, cut points for pain duration and subjective pain intensity were set. The former was set at 90 days and the latter at six or higher[11,12]. Statistical analysis included descriptive analysis of mean, minimum and maximum values and standard deviation (SD). For comparisons a combination of non-parametric (Mann Whitney-U test) and parametric (Independent samples T-test) were used. Statistical analysis was conducted using SPSS version 16.

## Results

Out of a cohort of 83 participants, 70 reported symptoms. Eighty three percent of the symptomatic participants indicated that they had consulted a health care practitioner regarding their neck pain. The mean subjective pain level was 4.46 (with minimum and maximum values ranging between 1 and 10) and the mean number of pain days in the preceding 12 months was 156 (with minimum and maximum values ranging between 1 and 365 days). A mean value of 5.49 on the BAI was observed, which was well below the 21-point index threshold required for anxiety to be considered clinically relevant (table 1).

**Table 1:** Numerical Pain Rating Scale 101 (NRS-101) and Beck Anxiety Index (BAI) scores by pain duration and pain intensity.

		*BAI*		
		Mean	SD	p-value
*All subjects*		5,49	4,612	
*Pain duration*	0-90 days (n = 29)	5,10	4,271	0,497
	>90 days (n = 40)	5,65	4,886	
*Pain intensity*	NRS-101 ≤ 6 (n = 53)	4,91	4,563	0,064^ϕ^
	NRS-101 > 6 (n = 17)	7,29	4,413	

N = 69 for pain duration & N = 70 for pain intensity.

^ϕ ^Statistically insignificant according to independent samples T-Test.

We also observed a mean subjective pain score of 2.83 (SD 1.891) amongst the shorter duration sub-groups, where as in the longer symptom duration sub-group a mean value of 5.42 (SD 1.893) was noted. This was highly statistically significantly different (p = 0,000), however, anxiety levels were unaffected. Furthermore, when our data were stratified according to pain intensity, no statistically significant difference was observed between the resulting sub-groups (p = 0,064) (Table 1).

## Conclusion and perspectives

The symptomatic participants in our study had experienced a relatively high intensity of pain over a protracted period of time. Yet, they appeared not to be anxious about their pain. Within the descriptive confines of our study and remaining respectful of Occam's razor, we offer three perspectives:

Firstly, our observations may indicate that chronic and/or intense non-specific neck pain simply does not trigger anxiety in the manner reflective of serious disease, especially in socially functional individuals[2].

Secondly, the lowering of anxiety levels may be indicative of a coping strategy[5]. The subjects in our study, most of whom had consulted or were under the care of a health care practitioner, may have learnt to identify pain symptoms as non-threatening[13]. Pain sufferers cannot always reduce their pain intensity, but can psychologically confront the experience, thus gaining control over the pain in their daily lives.

Finally, our observations may illustrate an example of alexithymia. This condition, characterized by a lack of words for feelings to express anxiety, anger or sadness, is found in patients with a tendency of somatising and appears to express itself, in particular, in chronic pain sufferers[14]. The difficulty of an alexithymic person in identifying and describing feelings increases symptom reporting of somatic sensations as tension or pain, but reduces the expression of an emotion like anxiety.

At face value, our results appear to challenge the notion that neck pain is necessarily significantly associated with heightened levels of anxiety, even when such pain is of a longstanding and fairly intense nature. This is an interesting finding in the context of recent pronouncements by the Task Force on Neck Pain and Associated Disorders[12] where psychologic and social factors are considered important in neck pain outcome within the general and clinical populations. The current findings indicate that this may not always be the case. Thus, we encourage further investigations aimed specifically at determining the role of anxiety in the course of chronic neck pain sufferers in the general population.

## Abbreviations

MPS: Myofascial Pain Syndrome; TP: Trigger Point; BAI: Beck Anxiety Index; DS: Standard Deviation.

## Competing interests

The authors declare that they have no competing interests.

## Authors' contributions

CM was the primary investigator in this project and drafted the manuscript. AHL was a co-investigator, responsible for data collection, and participated in revisions of the manuscript. KKR contributed to the interpretation of the data and participated in revisions of the manuscript. JH secured funding for the study, was responsible of the overall supervision of the project, and participated in revisions of the manuscript. All authors read and approved the final manuscript.
